# Observations on the Diseases and Medical Practice of Boutan and Thibet; Being Part of a Paper Entitled "Some Account of the Vegetable and Mineral Productions of Boutan and Thibet"

**Published:** 1790

**Authors:** Robert Saunders

**Affiliations:** Surgeon at Boglepoor in Bengal; inserted in the Philosophical Transactions of the Royal Society of London


### VIII. *Observations on the Diseases and medical Practice of Boutan and Thibet; being Part of a Paper entitled "Some Account of the vege- "table and mineral Productions of Boutan and "Thibet; by Mr.* Robert Saunders, *Surgeon "at Boglepoor in Bengal;" inserted in the Philosophical Transactions of the Royal Society of London,* Vol. LXXIX. *for the Year* 1789, Part I.

OF the diseases of this country, the first that attracts our notice, as we approach the foot of the hills, is a glandular swelling in the throat, which is known to prevail in similar situations in some parts of Europe, and generally ascribed to an impregnation of the water from snow. The disease being common at the foot of the Alps, and confined to a tract of country near these mountains, has first given rise to the idea of its being occasioned by snow water. If a general view of the disease, and situations where it is common, had been the subject of inquiry, or awakened the attention of any able practitioner, we should have been long since undeceived in this respect. On the coast of Greenland, the mountainous parts of Wales

Wales and Scotland, where melted ſnow muſt be continually paſſing into their rivers and ſtreams, the diſeaſe is not known, though it is common in Derbyſhire, and ſome other parts of England. Rungpore is about one hundred miles from the foot of the hills, and much farther from the ſnow, yet the diſeaſe is as frequent there as in Boutan. In Thibet, where ſnow is never out of view, and the principal ſource of all their rivers and ſtreams, the diſeaſe is not to be met with; but what puts the matter paſt a doubt, is the frequency of the diſeaſe on the coaſt of Sumatra, where ſnow is never to be found. On finding the vegetable productions of Boutan the ſame as thoſe of the Alps in almoſt every inſtance, it occurred to me that the diſeaſe might ariſe from an impregnation of the water by theſe plants, or the ſoil probably poſſeſſing ſimilar qualities, the ſpontaneous productions of both countries, with very few exceptions, being ſo nearly alike. It, however, appears more probable, that the diſeaſe is endemial, proceeding from a peculiarity in the air of ſituations in the vicinity of mountains with ſuch ſoil and vegetable productions. I am the more inclined to think ſo, that I have univerſally found this diſeaſe moſt prevalent amongſt the lower claſs of people, and thoſe who are moſt expoſed to the unguarded influence of the weather, and various changes that take place in the air of ſuch ſituations. The primary cauſe in the atmoſphere producing this effect is, perhaps, not more inexplicable than what we meet with in the low lands of Eſſex and fens of Lincolnſhire. An accurate analyſis of the water uſed in common by the natives, where this diſeaſe is more or leſs frequent, and where it is not known in ſimilar expoſures, might throw ſome light on this ſubject.

This very extraordinary diſeaſe has been little attended to from obvious reaſons: it is unaccompanied with pain, ſeldom fatal, and generally confined to the poorer ſort of people. The tumour is unſightly, and grows to a troubleſome ſize, being often as large as a perſon's head. It is certainly not exaggerating to ſay, that one in ſix of the Rungpore diſtrict, and country of Boutan, has the diſeaſe.

As thoſe who labour moſt, and are the leaſt protected from the changes of weather, are moſt ſubject to the diſeaſe, we univerſally find it in Boutan more common with the women than men. It generally appears in Boutan at the age of thirteen or fourteen, and in Bengal at the age of eleven or twelve; ſo that in both countries the diſeaſe ſhows itſelf about the age of puberty. I do not believe this diſeaſe has ever been removed, though a mercurial courſe ſeemed to check its progreſs, but did not prevent its advance after intermitting the uſe of mercury. An attention to the primary cauſe will firſt lead to a proper method of treating the diſeaſe; a change of ſituation for a ſhort time, at that particular period when it appears, might be the means of preventing it.

The people of this happy climate are not exempt from the venereal diſeaſe, which ſeems to rage with unremitting fury in all climates, and proves the greateſt ſcourge to the human race. It has been long a matter of doubt whether this diſeaſe has ever been cured by any other ſpecific than mercury and its different preparations. In defence of the opinion of other ſpecifics being in uſe, it has always been urged that the diſeaſe is frequent in many parts of the world where it could not be ſuppoſed that they were acquainted with quickſilver, and the proper method of preparing it as a medicine. I muſt own that I expected to have been able to have added one other ſpecific for this diſeaſe to our liſt in the Materia Medica, being being informed that the difeafe was common, and their method of treating it fuccefsful; nor could I allow myfelf to think they were acquainted with the method of preparing quickfilver, fo as to render it a fafe and efficacious medicine. In this, however, I was miftaken.

The difeafe feems in this country to make a more rapid progrefs, and rage with more violence, than in any other. This is to be accounted for from the groffnefs of their food and little attention to cleanlinefs.

There is one preparation of mercury in common ufe with them, and made after the following manner: — A portion of alum, nitre, vermilion, and quickfilver, are placed in the bottom of an earthen pot, with a fmaller one inverted put over the materials, and well luted to the bottom of the larger pot. Over the fmall one, and within the large one, the fuel is placed, and the fire continued for about forty minutes. A certain quantity of fuel, carefully weighed out, is what regulates them with refpect to the degree of heat, as they cannot fee the materials during the operation. When the veffel is cool, the fmall inverted pot is taken off, and the materials collected for ufe. I attended the whole of the procefs, and examined the materials afterwards. The quickfilver had been acted on by the other ingredients, deprived of its metallic form, and rendered a fafe and efficacious remedy.

A knowledge of chemiftry has taught us a more certain method of rendering this valuable medicine active and efficacious; yet we find this preparation anfwering every good purpofe, and by their guarded manner of exhibiting it perfectly fafe. This powder is the bafis of their pill, and often ufed in external application. The whole, when intimately mixed, formed a reddifh powder, and was made into the form of pills by the addition of a plum or date. Two or three pills taken twice a day generally bring on, about the fourth or fifth day, a fpitting, which is encouraged by continuing the ufe of the pills for a day or two longer. As the falivation advances, they put a ftick acrofs the patient's mouth, in the form of a gag, and make it faft behind. This, they fay, is done to promote the fpitting, and prevent the lofs of their teeth. They keep up the falivation for ten or twelve days, during which time the patient is nourifhed with congee and other liquids. Part of this powder is often ufed externally by diffufing it in warm water, and and washing sores and buboes. They disperse buboes frequently by poultices of turnip tops, in which they always put vermilion, and sometimes musk. Nitre, as a cooler, is very much used internally by them in this disease, and they strictly enjoin warmth and confinement during the slightest mercurial course. Buboes, advanced to suppuration, are opened by a lancet, with a large incision, which they do not allow to close before the hardness and tumour are gone. In short, I found very little room for improving their practice in this disease. I introduced the method of killing quicksilver with honey, gave them an opportunity of seeing it done, and had the satisfaction of finding it successfully used by themselves before we left the country.

This happy climate presents us with but little variety in their diseases. Coughs, colds, and rheumatism, are more frequent here than in Bengal. Fevers generally arise here from a temporary cause, are easily removed, and seldom prove fatal. The liver disease is occasionally to be met with, and complaints in the bowels are not unfrequent; but the grossness of their food, and uncleanliness of their persons, would, in any other climate, be the source of of conſtant diſeaſe and ſickneſs. They are ignorant (as we were not many years ago) of the proper method of treating diſeaſes of the liver and other viſcera: this is, I believe, the cauſe of the moſt obſtinate and fatal diſeaſe to be met with in the country, I mean the dropſy. As the Rajah had ever been deſirous of my aid and advice, and had directed his doctors to attend to my private inſtructions and practice, I endeavoured to introduce a more judicious method of treating thoſe diſeaſes by mercurial preparations. I had an opportunity of proving the advantage of this plan to their conviction in ſeveral inſtances, and of ſeeing them initiated in the practice.

The Rajah favoured me with above ſeventy ſpecimens of the medicines in uſe with them. They have many ſorts of ſtones and petrifactions, ſaponaceous to the touch, which are employed as an external application in ſwellings and pains of the joints. They often remove ſuch complaints, and violent head-achs, by fumigating the part affected with aromatic plants and flowers. They do not ſeek for any other means of information reſpecting the ſtate of a patient than that of feeling the pulſe; and they confidently ſay that the ſeat of pain and difeafe is eafily to be difcovered, not fo much from the frequency of the pulfe as its vibratory motion. They feel the pulfe at the wrift with their three fore fingers, firft of the right, and then of the left hand; after preffing more or lefs on the artery, and occafionally removing one or two of the fingers, they determine what the difeafe is. They do not eat any thing the day on which they take phyfic, but endeavour to make up the lofs afterwards by eating more freely than before, and ufing fuch medicines as they think will occafion coftivenefs.

The many fimples in ufe with them are from the vegetable kingdom, collected chiefly in Boutan. They are, in general, inoffenfive and very mild in their operation. Carminatives and aromatics are given in coughs, colds, and affections of the breaft. The centaury, coriander, carraway, and cinnamon, are of this fort. This laft is with them the bark of the root of that fpecies of Laurus formerly mentioned as a native of this country. The bark from the root is in this plant the only part which partakes of the cinnamon tafte; and I doubt very much if it could be diftinguifhed by the beft judges from what we call the true cinnamon. The bark, leaves, berries, and ftalks of many fhrubs

ſhrubs and trees, are in uſe with them, all in decoction. Some have much of the aſtringent bitter taſte of our moſt valuable medicines, and are generally employed here with the ſame view, to ſtrengthen the powers of digeſtion, and mend the general habit. Their principal purgative medicines are brought by the Chineſe to Laſſa. They had not any medicine that operated as a vomit, till I gave the Rajah ſome ipecacuanha, who made the firſt experiment with it on himſelf.

In bleeding they have a great opinion of drawing the blood from a particular part. For head-achs they bleed in the neck; for pains in the arm and ſhoulder, in the cephalic vein; and of the breaſt or ſide, in the median; and if in the belly, they bleed in the baſilic vein. They think pains of the lower extremity are beſt removed by bleeding in the ancle. They have a great prejudice againſt bleeding in cold weather; nor is any urgency or violent ſymptom thought at that time a ſufficient reaſon for doing it.

They have their lucky and unlucky days for operating or taking any medicine; but I have known them get the better of this prejudice, and be prevailed on.

Cupping is much practifed by them : a horn, about the fize of a cupping glafs, is applied to the part, and by a fmall aperture at the other end they extract the air with their mouth. The part is afterwards fcarified with a lancet. This is often done on the back; and in pain and fwelling of the knee it is held as a fovereign remedy. I have often admired their dexterity in operating with bad inftruments. Mr. Hamilton gave them fome lancets, and they have fince endeavoured, with fome fuccefs, to make them of that form. They were very thankful for the few I could fpare them. In fevers they ufe the Kuthullega nut, well known in Bengal as an efficacious medicine. They endeavour to cure the dropfy by external applications, and giving a compounded medicine made up of above thirty different ingredients: they feldom or never fucceed in effecting a cure of this difeafe. I explained to the Rajah the operation of tapping, and fhewed him the inftrument with which it was done. He very earneftly expreffed a defire that I fhould perform the operation, and wifhed much for a proper fubject; fuch a one did not occur while I remained, and perhaps it was as well both for the Rajah's patients and my own credit; dit; for after having feen it once done he would not have hefitated about a repetition of the operation. Gravelifh complaints and the ftone in the bladder are, I believe, difeafes unknown here.

The fmall pox, when it appears among them, is a difeafe that ftrikes them with too much terror and confternation to admit of their treating it properly. Their attention is not employed in faving the lives of the infected, but in preferving themfelves from the difeafe. All communication with the infected is ftrictly forbidden, even at the rifk of their being ftarved, and the houfe or village is afterwards erafed. A promifcuous and free intercourfe with their neighbours not being allowed, the difeafe is very feldom to be met with, and its progrefs always checked by the vigilance and terror of the natives. Few in the country have had the difeafe. Inoculation, if ever introduced, muft be very general to prevent the devaftation that would be made by the infection in the natural way; and where there could not be any choice in the fubject fit to receive the difeafe, many muft fall a facrifice to it. The prefent Rajah of Thibet was inoculated, with fome of his followers, when in China with the late Tiſhoo Lama.

The hot bath is uſed in many diſorders, particularly in complaints of the bowels and cutaneous eruptions. The hot wells of Thibet are reſorted to by thouſands. In Boutan they ſubſtitute water warmed by hot ſtones thrown into it.

In Thibet the natives are more ſubject to ſore eyes and blindneſs than in Boutan. The high winds, ſandy ſoil, and glare from the reflection of the ſun, both from the ſnow and ſand, account for this.

I have dwelt long on this ſubject, becauſe I think the knowledge and obſervations of theſe people on the diſeaſes of their country, with their medical practice, keep pace with a refinement and ſtate of civilization which ſtruck me with wonder, and no doubt will give riſe to much curious ſpeculation when known to be the manners of a people holding ſo little intercourſe with what we term civiliſed nations.

